# On drawing a line through the spectrogram: how do we understand deficits of vocal pitch imitation?

**DOI:** 10.3389/fnhum.2015.00271

**Published:** 2015-05-15

**Authors:** Peter Q. Pfordresher, Pauline Larrouy-Maestri

**Affiliations:** ^1^Department of Psychology, University at BuffaloBuffalo, NY, USA; ^2^Department of Psychology, University of LiègeLiège, Belgium; ^3^Department of Neuroscience, Max Planck Institute for Empirical AestheticsFrankfurt, Germany

**Keywords:** poor-pitch singing, vocal imitation, music performance, musical deficits, singing assessment

## Abstract

In recent years there has been a remarkable increase in research focusing on deficits of pitch production in singing. A critical concern has been the identification of “poor pitch singers,” which we refer to more generally as individuals having a “vocal pitch imitation deficit.” The present paper includes a critical assessment of the assumption that vocal pitch imitation abilities can be treated as a dichotomy. Though this practice may be useful for data analysis and may be necessary within educational practice, we argue that this approach is complicated by a series of problems. Moreover, we argue that a more informative (and less problematic) approach comes from analyzing vocal pitch imitation abilities on a continuum, referred to as *effect magnitude regression*, and offer examples concerning how researchers may analyze data using this approach. We also argue that the understanding of this deficit may be better served by focusing on the effects of experimental manipulations on different individuals, rather than attempt to treat values of individual measures, and isolated tasks, as absolute measures of ability.

## Introduction

The past decade has witnessed a surge in research on individual differences in singing from a cognitive science perspective (for recent reviews see Pfordresher and Mantell, 2009; Dalla Bella et al., 2011; Tsang et al., 2012; Berkowska and Dalla Bella, 2013). A core issue within this research has to do with how to set the boundary that defines which singers are considered “accurate” or “good,” and which singers may be “inaccurate,” “poor,” “tone-deaf," et cetera (e.g., Pfordresher et al., 2010; Hutchins and Peretz, 2012; Hutchins et al., 2012; Berkowska and Dalla Bella, 2013; Dalla Bella, 2015). Typically, these categories are treated as discrete, although there is agreement that more than one kind of internal deficit may push an individual into the “poor” category (e.g., Hutchins and Peretz, 2012). The *standard practice* (by our assessment) in recent literature has been to use this grouping variable as one factor in an analysis of variance (ANOVA), which may be dichotomous (e.g., Pfordresher and Brown, 2007; Moore et al., 2008; Wise and Sloboda, 2008; Hutchins and Peretz, 2012; Berkowska and Dalla Bella, 2013) or may have three levels that include an intermediate category (e.g., Joyner, 1969; Killian, 1991; McCoy, 1997; Price, 2000; Demorest and Clements, 2007; Moore et al., 2007).

Although the use of discrete categories is expedient for statistical analysis and is cognitively efficient, we here suggest that the field may be better served by changing course. We propose an approach that evaluates how pitch imitation performance varies on a continuum, which we refer to as *effect magnitude regression*. We suggest that this approach may prove more informative than the standard approach, while avoiding some important complications. The article begins by discussing the complications, including the inherent potential for oversimplification that comes from the use of discrete categories, variability in how an individual may be assessed using different measures or tasks, and difficulty identifying separate groups from distributions of performance. We go on to demonstrate how two specific effects, first demonstrated using the standard practice, may be better evaluated using a regression-based approach that examines each effect on a continuum.

As a matter of terminology, we contextualize singing as one manifestation of a more general ability involved in the vocal imitation of pitch. As such, in this paper we will refer to “vocal pitch imitation” rather than “singing”, and will refer to individuals as having a “vocal pitch imitation deficit” (VPID) as opposed to “poor-pitch singers.” There are two reasons for the present terminology. First, the kind of singing tasks we address are those that involve replication (imitation) of a specific standard (which may be one pitch or a melody), and the analyses we present focus on vocal pitch accuracy. Thus, we are not addressing aspects of singing that bear on features other than pitch accuracy (e.g., rhythms, expressive nuances), nor are we addressing forms of singing that do not involve the intention to replicate a specific ideal pitch pattern (e.g., improvisation). Second, although the tasks we discuss here do involve singing, recent research suggests that most individuals who fail to match pitch while singing exhibit similar deficiencies when imitating spoken intonation (Mantell and Pfordresher, 2013).

## The Trouble with Dichotomies

Categories help simplify the complexity of stimuli in our environment, and as such the tendency to form categories and hierarchies of categories is prominent in human and animal cognition (e.g., Harnad, 2003; Ashby and Maddox, 2005). However, because categories simplify the physical environment, it is worth treating discrete categories of individuals with caution.

Whether or not categorization of individuals into groups constitutes over-simplification depends in part on how research is directed. Some scientific questions, for instance, may be oriented toward a better understanding of how the listener perceives subjectively “good” or “bad” quality singing (Sundberg et al., 1996, 2013; Vurma and Ross, 2006; Larrouy-Maestri et al., 2013b, 2014), or the performance characteristics that lead to self-assessment in discrete categories (e.g., self-declared “tone deafness,” Wise and Sloboda, 2008). In applied settings, categorization into groups may be necessary based on practical demands, as in the case of a choir director who must either accept or reject singers based on an audition. However, for those of us wanting to understand mechanisms of vocal imitation in their own right, we suggest that the tendency toward use of discrete categories is problematic.

The sense that individual differences in vocal imitation accuracy may not be as discrete as one might think has already been expressed in Dalla Bella and Berkowska (2009), who proposed different “phenotypes” of VPID based on the fact that individuals differ with respect to the inaccuracy they exhibit on various tasks. Along similar lines Hutchins and Peretz (2012) proposed different varieties of VPID based on whether participants show sensitivity to the timbre of a pitch they attempt to match. We see these directions as useful, but propose taking things a step further. Why not dispense with discrete categories altogether, and address how individuals differ on a continuum of performance?

Recent evidence suggests some basic problems with the reliability and validity of participant categories. Past attempts to determine frequencies of VPID have yielded highly variable estimates based on the criterion. Pfordresher and Brown (2007) reported a frequency of VPID around 15%, which has been replicated in some other research (Dalla Bella et al., 2007; Pfordresher et al., 2010), but has also been questioned on highly reasonable grounds (e.g., Hutchins and Peretz, 2012). As it stands, the frequency of individuals who fit the VPID category may range from 2 to 78% depending on which task or measure of production one uses (Berkowska and Dalla Bella, 2013; Loui et al., 2015).

Even when the research question directly involves the perception of discrete categories in vocal imitation performance, complexities arise. Take for instance the degree of deviation between a sung pitch and the target pitch one attempts to match that is necessary to categorize the note as an “error.” The standard measurement unit for musical pitch is cents, in which 100 cents is the difference between two adjacent pitch classes in equal tempered tuning (i.e., two adjacent keys on a standard keyboard). On the surface, any sung deviation outside a window of 100 cents around the target pitch (from 50 cents sharp to 50 cents flat) is an error. However, how errors are perceived may be a different matter. Some studies have found that deviations around 10–45 cents start to affect interval perception, suggesting better acuity than one might assume (Moran and Pratt, 1926; Ward, 1954; Rakowski and Miskiewicz, 1985; Rakowski, 1990; Vurma and Ross, 2006; Larrouy-Maestri et al., 2013a). However, other studies show greater tolerance for mistuning, between 50 and 70 cents (Lindgren and Sundberg, unpublished, quoted by Sundberg, 1979, 1982), or beyond a semitone (Rakowski, 1976; Siegel and Siegel, 1977; Burns and Ward, 1978; Halpern and Zatorre, 1979; Sundberg et al., 1996; Schellenberg, 2001). In addition, recent evidence suggests that listeners’ tolerance for mistuning is higher (a greater deviation is necessary to be heard as an error) for vocal timbres than for violin (Hutchins et al., 2012). Moreover, most of these studies have focused solely on short sequences or on the final tone of a sequence, leaving the role of surrounding context still under question (Larrouy-Maestri, unpublished doctoral dissertation). In conclusion, the way in which listeners categorize singers is still in itself an area of vigorous exploration, making the application of categories to research on production questionable.

## The Trouble with Measurement

The use of a broad array of tasks in a “battery” to measure musical abilities is longstanding (e.g., Seashore, 1919). Several standardized listening tests have been proposed (for a review, see Vispoel, 1999). Recently, researchers have proposed batteries that focus specifically on singing, often with the goal of identifying deficient individuals (e.g., the Sung Performance Battery, SPB, Berkowska and Dalla Bella, 2013; the AIRS test battery, Cohen et al., 2009; the Seattle Singing Accuracy Profile, SSAP, Demorest et al., 2015).

Generally speaking, a good test is designed to sample the domain of interest broadly. As such, existing batteries include a range of tasks including matching a single pitch, singing a pair of pitches that forms a melodic interval, imitating short unfamiliar melodies, and singing familiar melodies from memory (or via imitation). Details on these tasks can be found in the original papers; our focus for the present is on how tasks influence categorization of individuals. Performance on batteries is usually summarized through a composite score that incorporates all the tasks or a subset of the tasks that seem most predictive. What if different subscales lead to different classifications of individuals? Such is the case for singing. In a thorough investigation of the SPB, Berkowska and Dalla Bella (2013) found that a single performance measure could yield highly divergent counts of VPID individuals in tasks that involve single pitch matching (58%) vs. the imitation of a novel melody (78%). ^1^We reflect more on how different tasks influence distributions of performance scores in the next section.

Other factors related to performance batteries likewise can have a strong effect on performance, and thus on the percent of the population who may be said to exhibit VPID. Previous evidence suggests an advantage for vocal timbres over non-vocal timbres (Moore et al., 2007, 2008; Nikjeh et al., 2009; Hutchins and Peretz, 2012; Lévêque et al., 2012), and the timbre of one’s own voice over the voice of another individual (Moore et al., 2008; Hutchins and Peretz, 2012; Pfordresher and Mantell, 2014). Another potentially important factor may be whether one is accompanied while singing, manipulations of which have led to variable outcomes (Petzold, 1969; Goetze and Horii, 1989; Pfordresher and Brown, 2007; Wise and Sloboda, 2008; Hutchins et al., 2010; Nichols, unpublished doctoral dissertation). Finally, the extent of VPID is enhanced when participants are asked to imitate pitches that are far from the center of their range, as opposed to pitches that are more comfortable for them to sing (Sims et al., 1982; Small and McCachern, 1983; Green, 1989; Pfordresher and Brown, 2007; Hutchins et al., 2014).

Sources of variability like this are fairly standard in the testing of abilities. Indeed, it is to be expected that some tasks will be more complex than others. The problem as we see it emerges when one is required to commit to a specific score that acts as a dividing line separating groups. In such cases there must be some “ground truth” to a specific task, but what task is best? Ecologically minded individuals no doubt would favor the imitation of melodies over matching single pitches, but what melodies? Does it make sense to choose pitches that are as comfortable as possible for a participant when real-world singing may not offer such opportunities? Finally, even if a task can be identifiably ideal over others, there is another measurement related issue: the way in which one measures “accuracy.”

The most common way accuracy is measured is through the difference between sung and target pitch for individual notes after transforming each into cents (a scale in which 100 cents = 1 musical semitone, the difference in pitch between two adjacent piano keys). The magnitude of these deviations across trials can be used to assess the magnitude of one’s average deviation from target notes.

When singing accuracy is measured in this way, researchers typically use absolute values of each deviation score for each note, and then use the average value as a summary statistic. This practice is convenient and intuitively appealing because it disregards the sign of the deviation (sharp vs. flat), which is usually not of central interest, and prevents differently signed errors from canceling each other out (few if any would say that a person who sings half her pitches 100 cents sharp and the other half 100 cents flat a “good” singer). Nevertheless, this practice conflates a distinction that historically has been important in motor control (e.g., Schmidt and Lee, 1999), and has roots in statistical estimation (e.g., Winer et al., 1991). Specifically, the mean signed difference between a participant’s sung pitches and the various target pitches he or she attempts relates to what in motor control is called “accuracy,” but in an intuitive sense reflects a general response bias in performance. This is distinct from another measure of performance known as “precision” in motor control, which essentially reflects random variability in performance. These measures may reflect different components of what listeners experience as being “accuracy,” and may identify different varieties of VPID (cf. Pfordresher et al., 2010). Importantly, whereas an individual’s signed pitch deviations reflect accuracy in the formal sense used in statistics and motor control, taking a mean from the absolute values of these deviations reflects both accuracy, and precision.

Because of this, means for the absolute values of pitch deviation scores are higher in magnitude than the absolute value taken from the mean of signed deviation scores, as will be demonstrated in the next section. Thus, from the standpoint of using a single value as a criterion that separates accurate from VPID imitators, merely taking the absolute value can have significant influences. It is also worth noting that the practice of converting pitch deviation scores into dichotomous error scores (e.g., calling any sung note with a deviation outside a window of ±50 cents) is comparable to using the mean of absolute deviation scores.

Such measures are, of course, limited to singing tasks that involve specific target pitches (e.g., imitation of sung sequences immediately after hearing a model singer). However, when singing a familiar song from long-term memory, it may not be appropriate to assume that an individual is attempting to sing specific absolute pitch classes (though people may do this when recalling popular music, Levitin, 1994). For such tasks, one must focus on relative pitch content in production. This can be done by comparing pairwise differences in sung melodic intervals to matched pairwise differences in the target melody. In addition to the comparison of consecutive notes, one can also estimate the pitch stability (Dalla Bella et al., 2007), or the tonal center deviation (Hutchins et al., 2014; Larrouy-Maestri and Morsomme, 2014) by choosing specific notes along the tonal sequences. Such measures will reflect deviations in relative but not absolute pitch; a participant who imitates the interval C to G by singing D to A may be 100 cents sharp for each sung note but will be perfectly accurate with respect to relative pitch (both intervals are a perfect 5^th^).

For the purpose of this paper what matters most is the fact that measuring accuracy of intervals may lead to different categorization of individuals than categorization based on the accuracy of individual notes. The mean absolute value of interval deviation scores tends to be far lower than scores based on individual notes (Vurma and Ross, 2006; Pfordresher and Brown, 2007; Berkowska and Dalla Bella, 2013). For example, whereas Berkowska and Dalla Bella (2013) identified 78% of their samples as VPID for a melody imitation tasks when a 50-cents criterion was applied to notes (mentioned earlier), this count dropped to 34% of the sample when the same criterion and task was used but the measurement was of interval deviation rather than deviation of notes.

## The Trouble with Distributions

However performance is analyzed, the result is a distribution of scores that varies across individuals. The formation of discrete categories involves separating this distribution at some point. A simple way to segment a distribution of scores is when the scores cluster noticeably, by revealing a “break” in a frequency distribution, or (even better) two modes. A decisive outcome like this was published in a study on Absolute Pitch (the ability to label a musical pitch outside of a musical context). Although variability in performance on labeling tasks can be found for participants labeled as having or not having this ability, the distribution of scores on two similar tasks both revealed a dip in the frequency of scores, demonstrating a bimodal separation between the groups (Athos et al., 2007).

**FIGURE 1:**
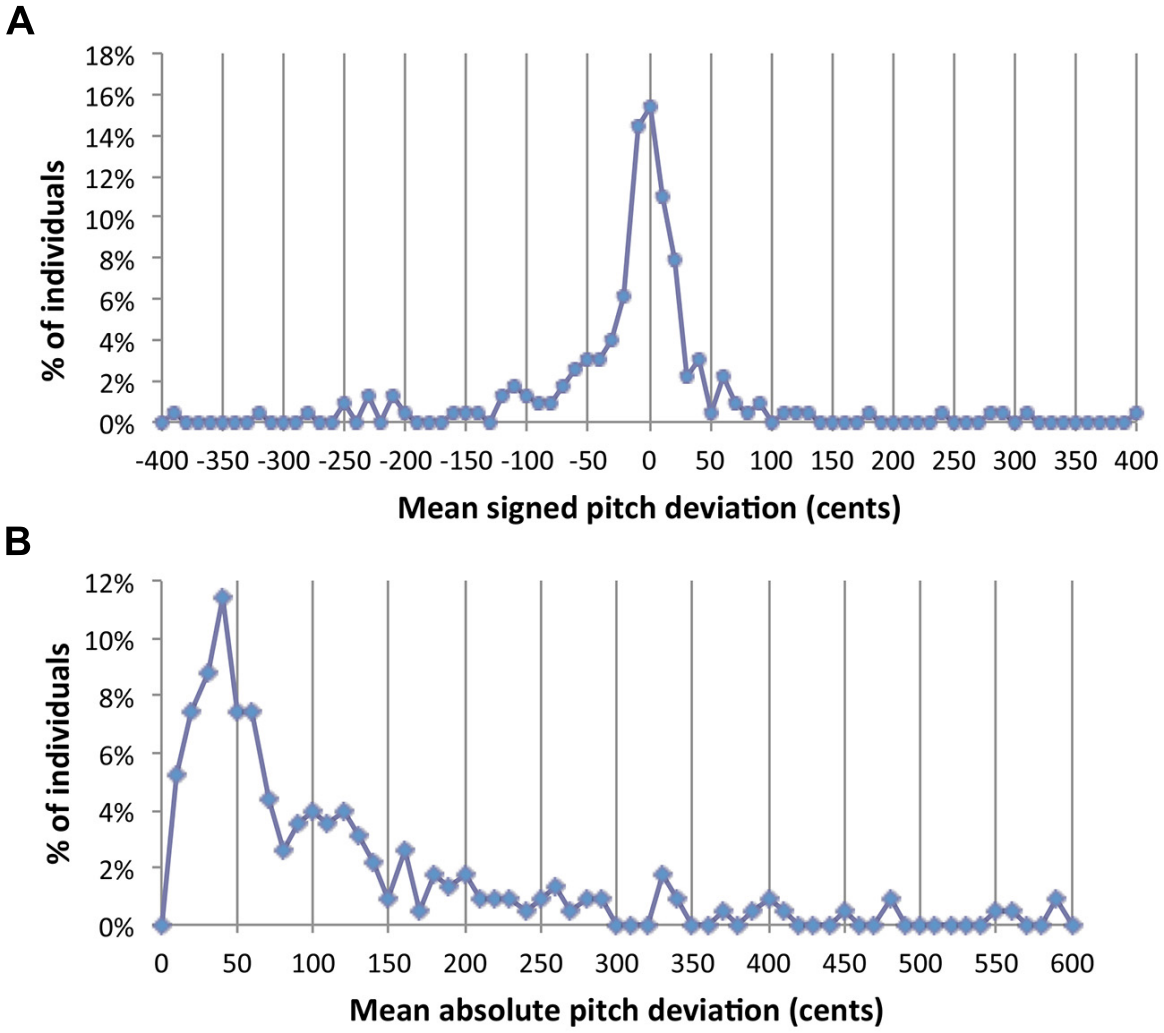
**Frequency distributions of scores for signed pitch deviations **(A)** and absolute pitch deviations **(B)** when averaged across a set of notes and trials for each individual.** Vertical lines highlight 50-cents increments on the X axis.

Where do singing abilities stand? **Figure 1** shows a distribution of singing accuracy measures from a large sample of data from our lab (*N* = 228), pooled across four published studies (Pfordresher and Brown, 2007, 2009; Pfordresher et al., 2010; Pfordresher and Mantell, 2014), and a sample of unpublished data (*n* = 26). All tasks involved the imitation of four-note isochronous, major-key sung sequences on a neutral syllable (/da/). The complexity of sequences included here varied from patterns including a single repeated pitch, to patterns with four distinct pitches (ranging within a perfect 5th). Both panels show frequency histograms relating to the deviation (in cents) of individual sung pitches from their respective target pitch in the sequence. Frequencies come from averages for each participant across all sung notes and trials. Data in **Figure 1A** come from the mean for each subject (across tasks) of the signed difference between sung and target pitches, whereas **Figure 1B** plots the mean of absolute differences for each subject. As mentioned in the previous section, means from absolute differences are typically larger in magnitude than signed differences.

Two points emerge from this analysis. First, neither panel shows a clear division between groups. There is no hint of a second mode or a clear gap in the frequency histogram. Second, the distributions here replicate the problem discussed in Berkowska and Dalla Bella (2013). If VPID singers are said to be those whose mean signed deviation is greater than +50 or less than -50, the percent of VPID singers comes out to be approximately 32%. However, taking the absolute value for each measured score makes this figure surge to 59% using the same criterion (an absolute value of 50 cents). Thus, differences in vocal imitation do not represent the clear distinction that was found for AP abilities. To be fair, most human abilities are continuously distributed, leaving the designation of a cutoff criterion up to statistical principles (e.g., two SD from the mean) or practical considerations (discussed before). However, the joint effect of the present complication along with issues brought in the previous section casts serious doubts on how effectively researchers can establish fixed and general cutoff criteria.

The second problem with distributions of this sort is statistical. A major convenience in dichotomizing groups is that “group” can be treated as a two-level factor in an ANOVA. Interactions of experimental manipulations with this dichotomous factor can be dealt with straightforwardly in simple effects analysis and interaction contrasts. However, a major assumption of ANOVA is homogeneity of variance and ANOVA is less robust to violations of this assumption than to violations of other assumptions (Keppel and Wickens, 2004). Using the present data as a paradigmatic example, the ratio of variances across groups (the metric used for the *F-max* test for homogeneity) is many orders of magnitude when using either a 50 or 100-cents criterion for either measure plotted in **Figure 1** (See **Table 1**). The smallest ratio obtained is 32.6 (for a criterion of 100 cents applied to the data of **Figure 1B**), and the largest is 202.7 (criterion of 50 cents applied to the data of **Figure 1A**). Under ideal circumstances this ratio should be 1 (perfect homogeneity) and critical values for the *F*-max statistic (which one does not want to exceed) are on the order of 1–3. Based on such extreme differences, the convenience of dichotomizing groups for use in ANOVA may be offset by questions of statistical validity.

**Table 1:** Group statistics based on different criteria for data plotted in **Figure 1**.

Type of measure	Signed deviation		Absolute deviation	
Type of criterion	50 cents	100 cents	50 cents	100 cents
M accurate	15.48	24.03	27.22	42.82
M VPID	188.66	267.75	173.87	233.37
% VPID	32%	19%	60%	38%
s^2^ Accurate	144.22	516	179.23	653.87
s^2^ VPID	29,229.47	31,701.43	19,659.14	21,333.46
Ratio	202.66	61.44	109.69	32.63

Issues summarized in this section are not uncommon in the behavioral sciences, and on their own are not insurmountable. With respect to heterogeneity of variance, for instance, a log transformation of the present data for instance remedies the problem (e.g., variance ratio for 50-cents criterion, absolute deviation becomes 1.23). For us, however, the problems detailed here add to the series of complications for standard practice that we have summarized so far. This brings us to our recommendations for a possibly more fruitful way to understand VPID.

## A New Way Forward

We suggest an alternate approach we refer to as *effect magnitude regression* that is designed to measure how the strength of an experimental effect varies across individuals based on each person’s overall accuracy. Specifically, we propose using measures of pitch imitation performance in a continuous rather than discrete way, as a predictor (X) variable. The Y variable is then a measurement of some experimental effect defined for each individual on this continuum. In this context, the X variable is the same kind of measure that may be used in a performance battery, cited earlier. The critical difference here is that the analysis focuses on the continuum of *X*-values, rather than using a specific value as a cutoff criterion. The Y variable measures the magnitude of an experimental effect for each individual and thus involves the kind of treatment variance that can also be measured in an ANOVA. In addition to avoiding conceptual and statistical problems, we think this approach more genuinely reflects the distribution of individual performance (continuous, rather than bimodal) and that it offers more information about the nature of individual differences.

Although this approach is not dominant in the literature, examples exist. For instance Granot et al. (2013, Figure 3) report a regression in which a predictor variable related to musical sophistication (OOT), is correlated with a Y variable measuring the difference in singing performance between imitating a live model (audio and visual) and the imitation of a recording (audio only). Participants scoring higher on the sophistication measure exhibited a larger advantage for the live model (i.e., a larger raw effect size), and thus a larger *Y*-value. Of course, the authors could have subjected the same data to an analysis that followed the standard practice. Such an analysis would have involved separating participants into dichotomous “low” and “high” sophistication groups and enter that as a quasi-experimental factor into an ANOVA or *t*-test. However, we think that the analysis reported by the authors is both more valid and more informative.

It is important to note that the effect magnitude regression we propose here is distinct from simpler bivariate regression that is reported in many papers (e.g., Dalla Bella et al., 2007, 2009; Hutchins and Peretz, 2012; Larrouy-Maestri et al., 2013b). Whereas bivariate regression can address relationship of two variables, it cannot speak to the influence of some manipulation (Y), as a function of some predictor variable (X). Ultimately we see bivariate regression and effect magnitude regression as complementing each other, as we will discuss in more detail later.

We now present two examples of effects from previous data sets that were originally presented using a dichotomous “group” variable ANOVA (the “standard” approach). These examples illustrate the possible pitfalls of the standard approach and demonstrate the proposed analysis using effect magnitude regression.

### Example 1: Effect of Chorused Feedback on Singing

The first example we choose concerns the effect of singing along with the correct melody (chorused, or augmented feedback), vs. singing solo (normal feedback), as reported by Pfordresher and Brown (2007, Experiment 1). Contrary to our initial intuitions, accuracy among VPID participants diminished rather than improved when these individuals experienced chorused as opposed to normal feedback, whereas the opposite was found for accurate imitators. This result suggests that VPID participants may not be able to generate an appropriate “efference copy” of their motor plan in order to minimize error between the additional feedback (the “chorus”) and feedback from oneself (Wolpert and Kawato, 1998; Pfordresher, 2011). Other studies have reported designs that partly replicated this procedure, but did not replicate the result. Hutchins et al. (2010) reported a null effect of chorused feedback when comparing congenital amusia with normal controls, and Wise and Sloboda (2008) reported improvement from chorused feedback among individuals who self-identify as “tone deaf.” Taken together, one wonders whether the initial effect was an artifact, or some kind of selective interaction based on how one defines group differences.

**FIGURE 2:**
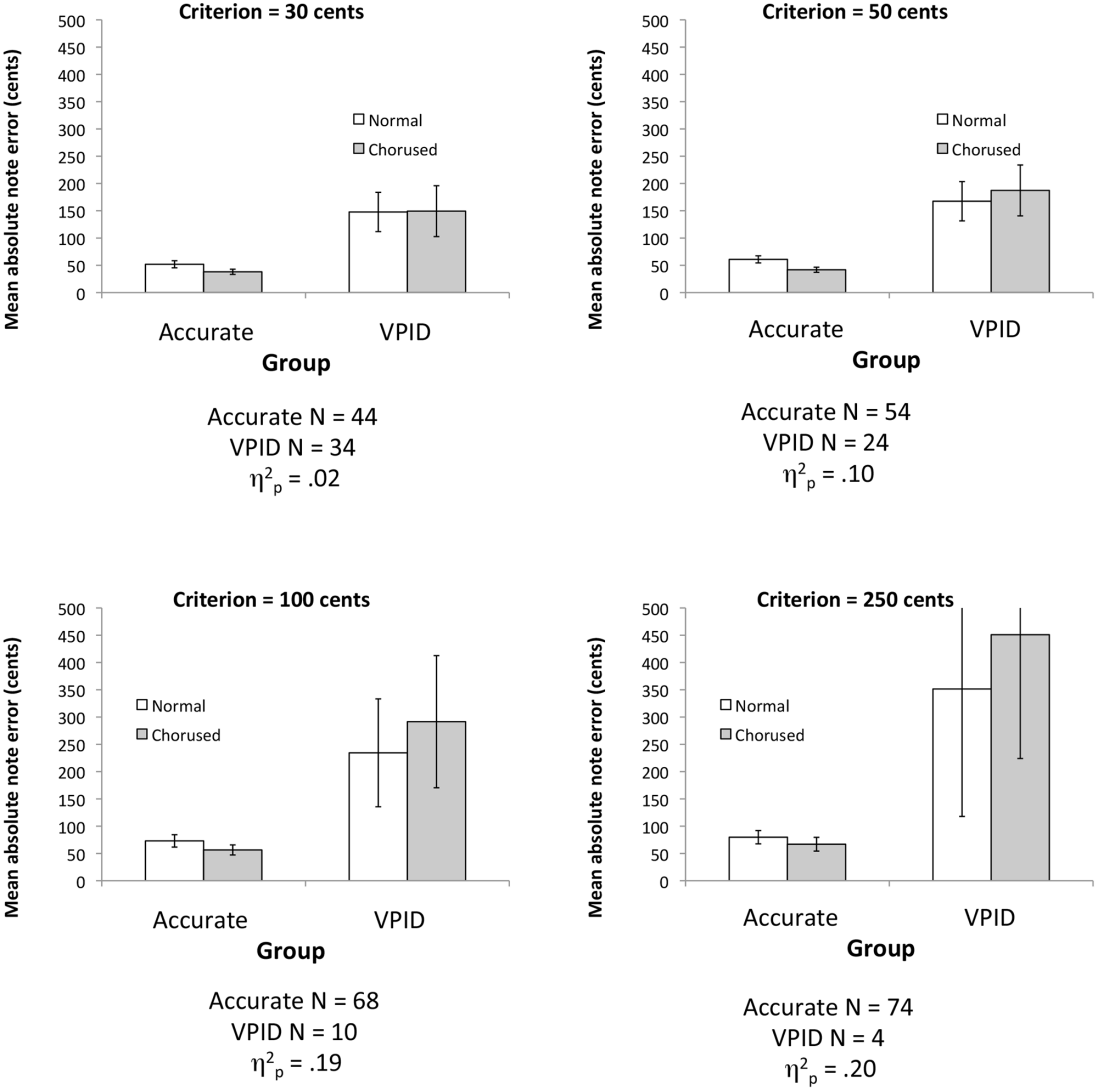
**Plots of the group × feedback interaction effect using data from Pfordresher and Brown (2007) based on different criteria for separating groups.** Error bars display 95% confidence intervals. Note that the upper error bars in the lower-right plot extend beyond the visible boundary of the Y-axis, and are equal in size to the lower error bars.

A re-analysis of the original data set reveals the effect to be highly sensitive to where one sets the criterion for dividing groups, which may partly account for variability across studies. **Figure 2** plots the interaction of group with feedback (normal or chorused) using four different criteria for distinguishing groups. More “liberal” criteria categorize more participants as VPID, and use a lower pitch deviation score as a cutoff. By contrast, more “conservative” criteria lead to fewer participants being categorized as VPID. As in Pfordresher and Brown (2007), we here used signed pitch deviation scores for separating groups. As can be seen, for more liberal thresholds (which categorize more participants as inaccurate), the interaction is negligible in its effect size, ^2^ whereas the interaction becomes increasingly large in its statistical effect size and visually salient when a more conservative criterion is used. Thus, this interaction may be particularly sensitive to the kind of criterion one uses.

**Figure 2** illustrates another important factor: sample size. More conservative criteria lead to smaller VPID samples, making the two groups highly imbalanced. These differences in sample size likewise influence the precision of estimation. The error bars in **Figure 2** show 95% confidence intervals (the original paper plotted SE), which are strongly influenced by sample size, given that smaller sample size increases both SE of the mean and also the criterion value of *t*. As such, when the interaction emerges, we also find a dramatic expansion of confidence intervals among inaccurate singers, suggesting that sample statistics lack reliability. This statistical factor, along with sensitivity to criterion, suggests that the ANOVA approach used in the original paper may not be ideal.

**FIGURE 3:**
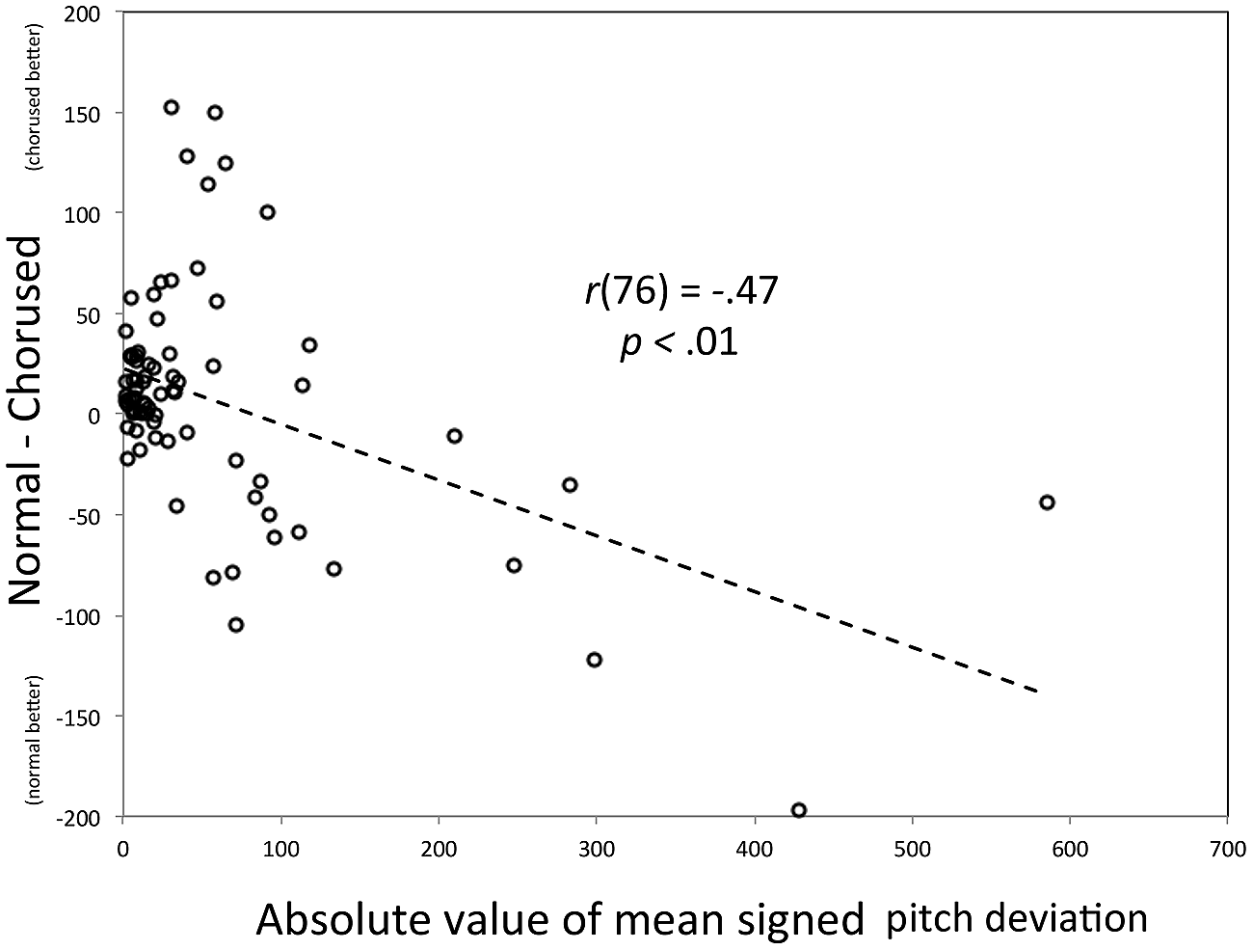
**Scatter plot of the relationship between the absolute value for the mean signed pitch deviation (X) and the difference in mean absolute pitch deviation across Chorused and Normal auditory feedback conditions (Y).** The individual participant is the parameter.

As such, we turn to effect magnitude regression (the “new way forward”) as an alternative way to explore the same effect. In this analysis, effect magnitudes (Y) were computed for each participant by using the difference in mean absolute pitch deviation scores between chorused and normal feedback (deviation_normal – deviation_chorused), with positive values indicating an advantage for chorused feedback. As can be seen in **Figure 3**, *Y*-values cluster at values slightly above zero (*Y*-intercept = 23 cents, a slight advantage for chorused feedback) for more accurate imitators, and grow increasingly negative for VPID participants, indicating worse performance for chorused than normal feedback. For consistency with the previous analysis (**Figure 2**), the X-axis is the absolute value of mean signed error for participants. We focused on this measure because it was used to separate groups in the original paper; a similar (though weaker) relationship emerges if the X-axis is replaced by the mean of absolute pitch deviations. This figure clearly shows the gradual increase in effect size across participants. Moreover, the regression parameters can be used to estimate the point at which the chorused feedback starts to impair performance. In this particular experimental context, the limit corresponds to approximately 100 cents deviation score. It is worth noting that most of the variance contributing to the regression lines is found among the VPID participants, thus underscoring our argument that treating this group as a single category is overly limiting.

### Example 2: The Attracting Effect of One’s “Comfort Pitch”

Whereas Example 1 involves two conditions and therefore the simplest kind of ANOVA effect (a 2 × 2 design), many experimental effects involve more complex patterns defined across multiple conditions. Thus, Example 2 is designed to show how effect magnitude regression can be extended to more complex designs. We turn to an effect (also reported in Pfordresher and Brown, 2007, in Experiment 2) that involves a pattern of results that is defined across five experimental conditions. Participants first generated a single pitch that they considered to be most comfortable for them. Then participants imitated four-note monotone sequences comprising pitches that were above, below, or equal to their comfort pitch. Analyses of signed pitch deviation scores suggested an attractor effect of the comfort pitch among VPID participants: target notes higher than one’s comfort pitch were sung “flat,” whereas target notes lower than one’s comfort pitch were sung “sharp” (for a similar recent result, see Hutchins et al., 2014).

**FIGURE 4:**
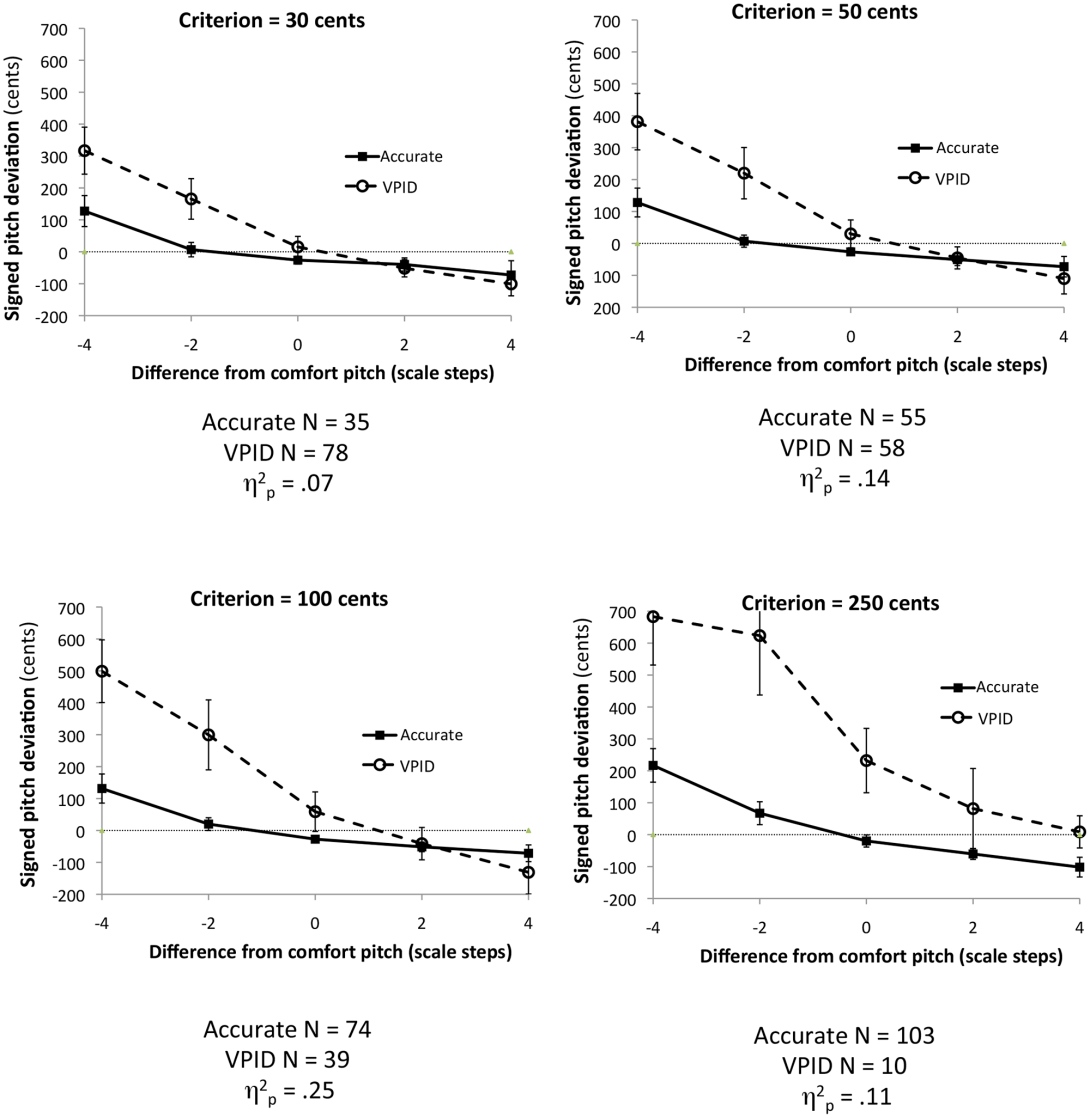
**Plots of the interaction between group and the difference between imitated and comfort pitch using data from Pfordresher and Halpern (2013), based on different criteria for separating groups.** Error bars display 95% confidence intervals.

The original effect was reported with a sample size that was too small to complete the kind of analysis reported in Example 1 (*N* = 14). Thus, we re-analyzed data from a larger sample (*N* = 135) reported in Pfordresher and Halpern (2013). That study used the same design as Pfordresher and Brown (2007, Experiment 2), although Pfordresher and Halpern did not test the attracting effect of one’s comfort pitch. **Figure 4** shows four analyses based on dichotomous groups, using the same method for dividing groups as in Example 1. As can be seen, although the interaction is apparent for each criterion (and is statistically significant in each case), the effect size of the interaction increases as the criterion becomes more conservative, up to a criterion of 100 cents. Then the effect diminishes again for the 250 cents criterion, probably because many participants classified as “accurate” in this analysis are showing the attractor effect. The apparent non-linearity of the change in effect size with criterion level leads one to wonder what the effect may look like on a continuum.

**FIGURE 5:**
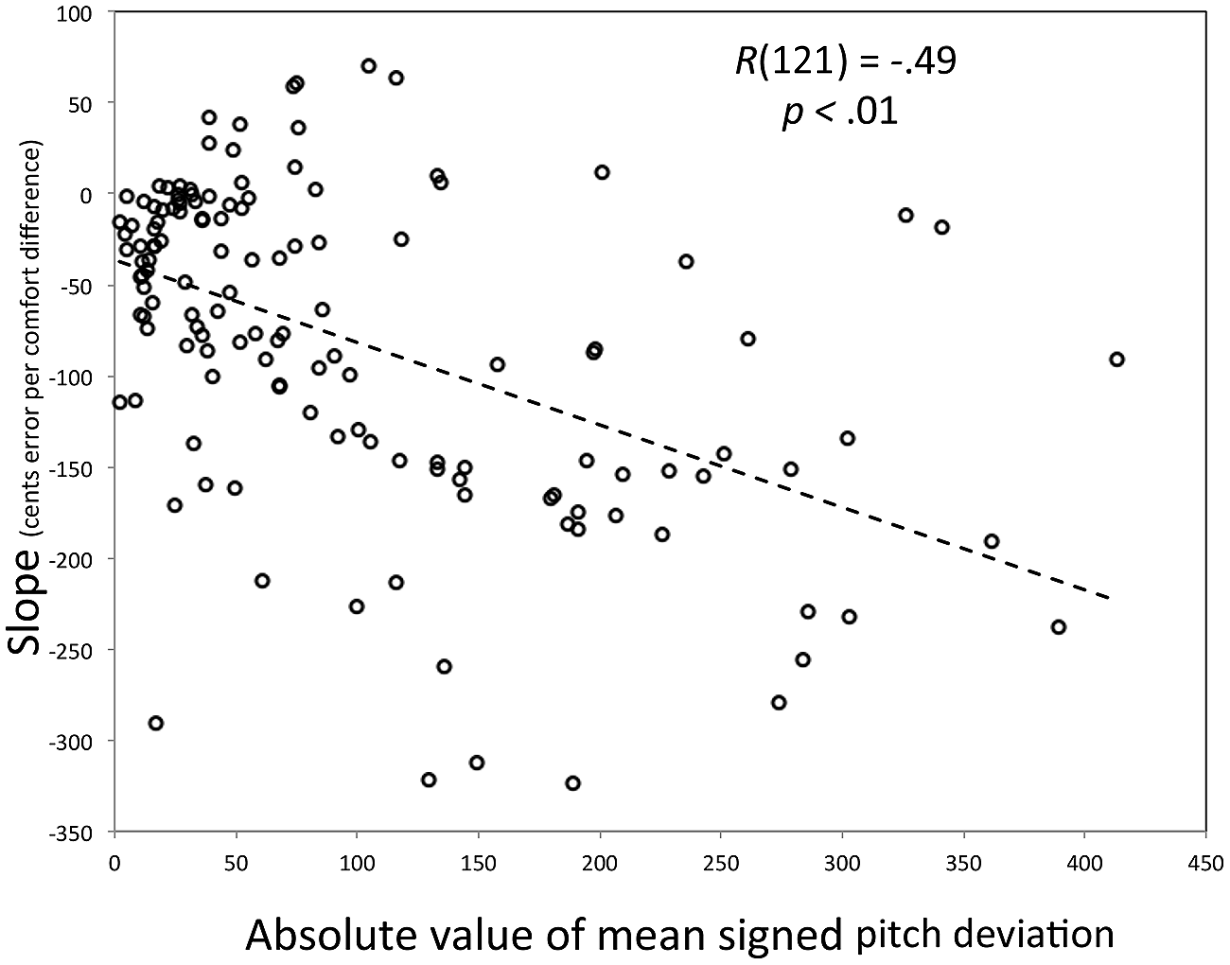
**Scatter plot of the relationship between the absolute value for the mean signed pitch deviation (X) and the slope relating difference from comfort pitch to signed pitch deviation in imitation (Y).** The individual participant is the parameter.

For this analysis, we defined effect magnitudes (Y) by computing a linear regression across trials for a single participant (i.e., regressing signed pitch deviation on the difference between target and comfort pitches), using the slope of that regression as an estimate of how strongly attracting the comfort pitch is. The more negative the slope, the stronger the attracting effect. As can be seen in **Figure 5**, most participants show an attracting effect, with the few positive slopes being small in size (≤75 cents). More important, as singers grow increasingly inaccurate, the attractor effect increases in size.

## Evaluation of Effect Magnitude Regression

We propose that effect magnitude regression allows one to see in more detail how a specific experimental effect is reflected across a continuum of vocal imitation performance, while avoiding the problematic issue of how one may categorize participants into discrete groups. This leads to a re-conceptualization of vocal imitation “deficits” as being graded rather than discrete (a point we will address further in the next section). However, the regression-based approach raises two new concerns. Ultimately, neither concern ends up posing a major problem for the approach although researchers should take care to address each when analyzing a given data set.

**FIGURE 6:**
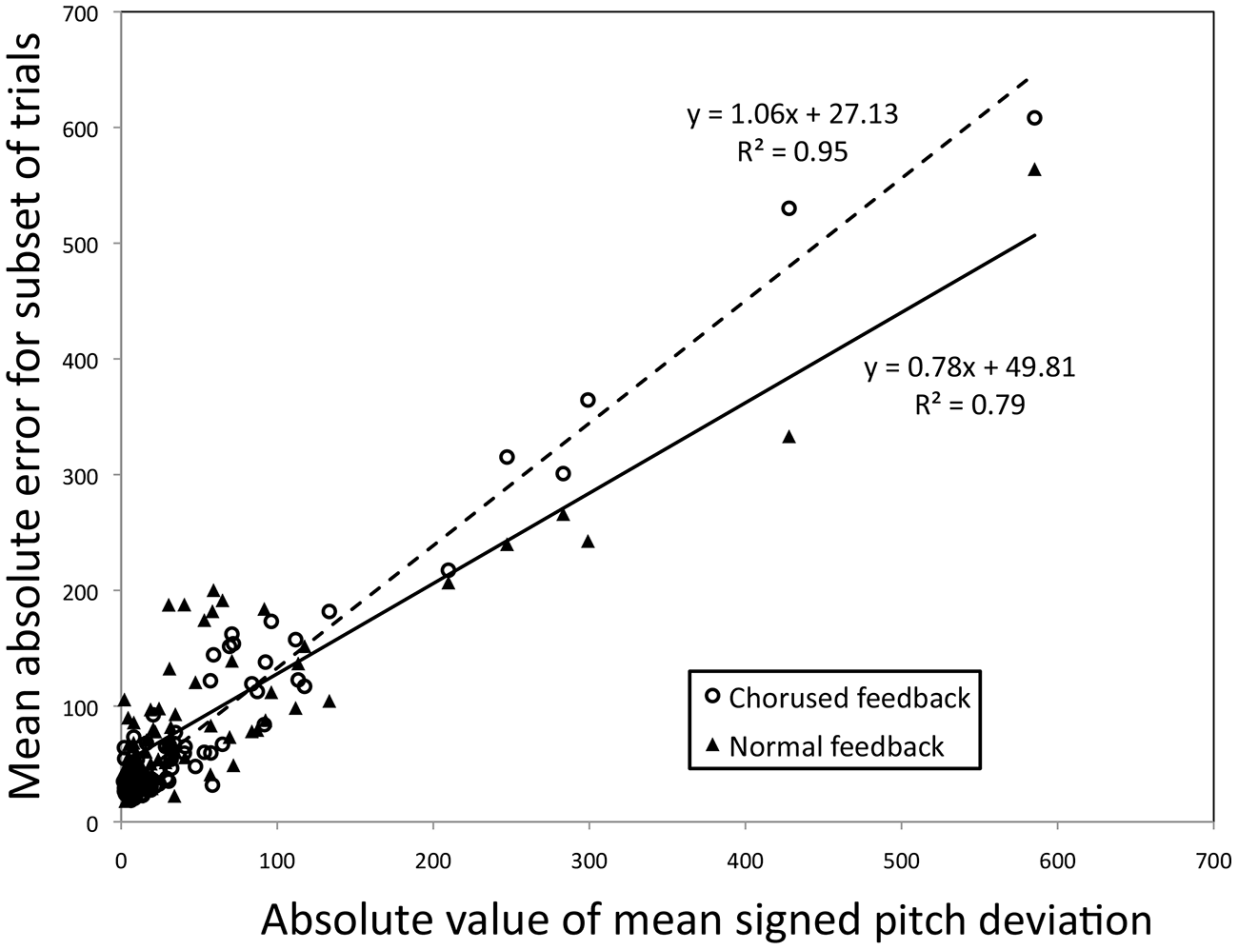
**Bivariate correlations between the absolute value for the mean signed pitch deviation (X) and the two subsets of the data leading to the difference between Chorused and Normal auditory shown in **Figure 3** (Y).** The individual participant is the parameter. Note that each participant yields two identical X-values but not two identical Y-values.

The first issue has to do with ambiguity, which was mentioned earlier, and relates to the measure of effect size (Y). Consider the use of difference scores, as in Example 1. It is unclear based solely on **Figure 3** whether the increase in Y with X is actually due to the difference between feedback conditions, or may be attributable solely to one condition (with the relationship between X and Y being constant for the other). As mentioned earlier, the simplest solution to this issue is to complement the effect size regression with simple bivariate regressions based on the scores being used in the effect (see Pfordresher and Mantell, 2014, for an example of this application). **Figure 6** shows such a plot for the effect of feedback from Example 1. Consistent with the regression based on difference scores, the regression line associated with chorused feedback has a steeper slope (1.06) than the regression line associated with normal feedback (0.78), although both lines yield significant fits (*p* < 0.01 for each). In this respect, one can assess the relative contribution of each association. Of course, the second example above is more unwieldy to plot, being based on five treatment conditions. In such cases, statistics for individual conditions (parameters and *r*^2^-values) may be better positioned in a table.

The second issue has to do with collinearity. Although it is possible (and arguably ideal) to have an X variable that is independent of measures contributing to Y (e.g., a separate screening task), that is not the case for the examples detailed here. For instance, in Example 1 (just discussed), each variable contributing to difference scores that make up the Y variable is a subset of all the trials that lead to a participant’s X score. As such, one would expect a strong relationship for the bivariate regressions shown in **Figure 6** (though a difference in slopes is not a foregone conclusion). Does a similar collinearity cloud the interpretation of effect magnitude regression?

We used Monte Carlo simulations to address whether any inherent dependency between X and Y exists for the effect magnitude regression approach. First, we randomly sampled absolute pitch deviation scores from a Beta distribution using the Matlab function “betarnd.” This distribution mimics the kind of skew one sees in actual data (e.g., **Figure 1B**). For our simulation we set parameters that led to a distribution that maximized resemblance to data: a mode at 75 cents, and a tail that extended to approximately 600 cents.

**FIGURE 7:**
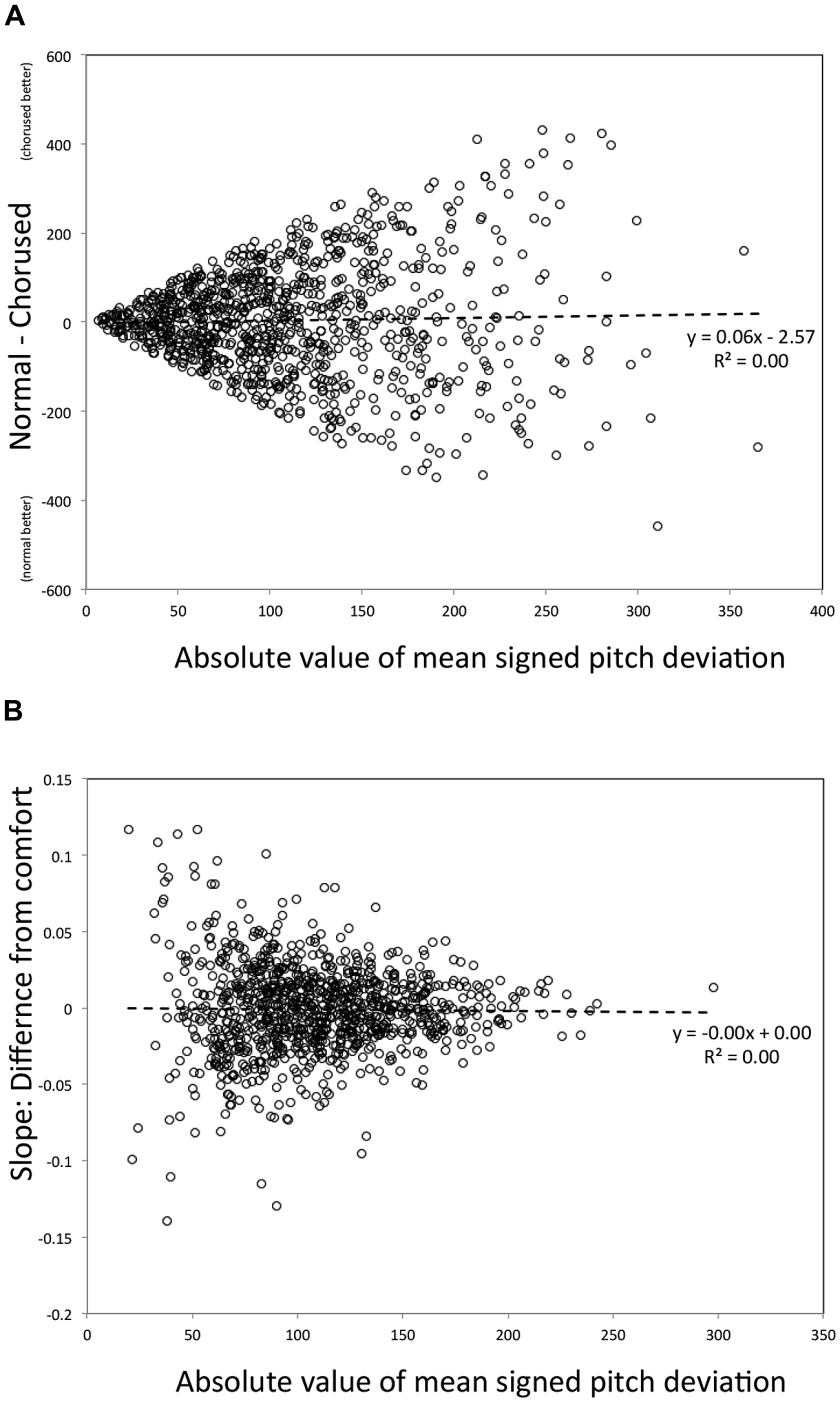
**Scatter plots of data from Montecarlo simulations of the regression plotted in **Figure 3 (A)** and **Figure 5** (B)**.

The simulation of Example 1, shown in **Figure 7A**, was done by sampling data points at random and pairing independent samples. These randomly selected pairs were used to simulate an effect of feedback one might find if the true effect size in the population was zero; any apparent effect would thus be an artifact resulting from how the regression was constructed. One thousand such pairs were generated randomly. *Y*-values in **Figure 7A** are thus differences between paired values, and X is the mean value for each pair. As can be seen, the slope relating the average across conditions to their difference is zero; thus the relationship reported in Example 1 would not have emerged simply by chance.

However, it is also worth noting that heterogeneity of variance, earlier discussed as an issue pertaining to standard ANOVA designs (see *The Trouble with Distributions*) remains in the data, here manifested in the fanning out of scores around the regression line as X increases. As a result, a mean of all *Y*-values taken from the left side of the X-axis would have a substantially lower variance than a mean of *Y*-values from the right side of the X-axis. Why does this happen? Due to the fact that the beta distribution (like mean absolute pitch deviation) is bounded at zero, difference scores leading to means near zero (left side of the plot) are restricted in range relative to points leading to high average *X*-values. Although this is a problem worthy of concern (which should be assessed empirically for any data set), by our understanding the problem is not as damaging within regression as it is within ANOVA for the current research context. Whereas in ANOVA, variance heterogeneity can lead to false positives (Keppel and Wickens, 2004), in regression the problem typically reduces the strength of association (Cohen and Cohen, 1983, pp. 128–129). More important, the regression analysis does not depend on comparisons between two portions of this regression line, but instead focuses on the sign and magnitude of the slope relating predictor and outcome variables, and this slope critically is not confounded by the nature of the measurement (unlike ANOVA, in which heterogeneity of variance can increase the likelihood of a type-I error).

We conducted a similar analysis to address whether the effect magnitude regression for Example 2 was spurious. For this simulation we used a Gaussian distribution (in Matlab, “normrnd”) with a mean of zero and standard deviation of 50, to approximate the distributional properties of signed pitch deviation scores (**Figure 1A**). We generated 1,000 random samples for each of the five conditions, and from these samples computed 1,000 regression lines using the *X*-values shown in **Figure 4** (with the five values in each regression being grouped arbitrarily). **Figure 7B** shows the regression of slope values (Y) on the average of absolute value of the mean across these conditions (X). Again, the resulting slope is zero, unlike what we found for Example 2. Heterogeneity of variance is apparent again, though it appears to be reduced relative to the Example 1 simulation, with a more consistent spread of scores around the regression line across the X-axis. In this case, variances are constrained for high values of X (large mean error). This happens because when mean deviations are high, extreme values of one sign tend to be counterbalanced by the opposite sign. Again, the critical point is that the slope from this simulation does not resemble the relationship found in Example 2.

## Implications

Several new functional models of the underlying system have accompanied the recent surge in empirical studies of vocal pitch imitation (e.g., Berkowska and Dalla Bella, 2009; Dalla Bella et al., 2011; Hutchins and Moreno, 2013). Following the standard cognitive framework, these models have proposed that vocal pitch information comes from the interaction of several basic functions that connect to form a network (Pfordresher et al., 2015). The functions themselves are represented as modules (though without an explicit commitment to modularity in the classic sense), the implication being that specific deficits may result from the malfunctioning of selected modules. Given that we here suggest a new approach to conceptualizing VPID, one may wonder if we are by association suggesting a move away from these kinds of models.

As we see it, the proposals made here possibly argue for a rethinking of how deficits are manifested in a particular architecture, but not necessarily the structure of that architecture itself. To use a simple example, consider the Linked Dual Representation model (Hutchins and Moreno, 2013). In this model, pitch perception links directly to both motor control and a symbolic representation of pitch (three basic functions). The symbolic pitch representation further connects to motor control. Thus, vocal motor control may be guided by pitch perception or a symbolic pitch code. Hutchins and Moreno (2013) suggest that much of VPID may be based on a breakdown of the connection between pitch perception and motor control with a spared connection from pitch perception to the symbolic code (resulting in accurate performance on perception tasks). Based on the approach advocated here, it may be possible to re-conceptualize deficits in this model as being based on the strength of the connection, involving both the precision of connections between perception, and production (how variable the link from one to the other is) and their accuracy (whether there is a systematic bias in connecting two components).

Another subtle, though important, implication has to do with the use of terms. Throughout this paper we fall back on standard terminology that is in itself dichotomous (“accurate” vs. “VPID” individuals), while at the same time arguing against the use of dichotomies. Here we run into the problem of linguistic convenience: it is far easier to talk in this dichotomous way than to refer to differences in degree. Ultimately, though we have indulged in dichotomous language we think that the two terms we use represent poles on a continuum, with individuals generally falling somewhere between the extremes.

Finally, the proposed approach focuses on effects of experimental manipulations and supports the use of different conditions to examine singing accuracy. Concretely, this approach moves away from the categorization based on a single task and is more akin to diagnosis tools used in other domains. For instance, in language disorders, the identification of dyslexia and the decision of therapeutic lines are based on the combination of different conditions (i.e., tasks) proposed in batteries (or tasks specifically chosen across different batteries). In other words, the evaluation is not simply based on the addition of performance scores at the different tasks but on the observation of contrasted conditions. Regarding vocal imitation deficits, we believe that our “new way forward” represents a way to follow that direction. Therefore, this approach could inspire statistical analysis (i.e., to examine the experimental effects themselves) of existing singing batteries in order to identify specific vocal imitation deficits.

Although the regression approach advocated here does ultimately fall back on the use of a single measure to function as a predictor (X) variable, the stakes associated with the choice of this measure are not as great (we think) as when a measure is used for categorization. Consider the two measures plotted in **Figure 1**. A researcher who uses one measure or the other to form discrete categories may yield substantially different group compositions based on their choice of score, as well as their choice of task (Berkowska and Dalla Bella, 2013). By contrast, the regression-based approach is likely to be less sensitive to such differences. Regression lines may differ slightly based on the choice of an *X-*variable, but the significance and direction of an effect (which is the most appropriate focus at this stage of the research) is less likely to vary. In keeping with this it is worth mentioning that the significance and direction of effect for both examples shown here remains the same if we use the mean of absolute pitch deviation scores (as opposed to the measures shown here) as the *X*-variable.

## Conclusion

In this article, we have advocated an approach to studying VPID (a.k.a. poor-pitch singing) that focuses on individual differences across a continuum, rather than categorizing individuals into discrete categories based on success or failure at vocal pitch imitation. Conceptually, our proposal involves a change in perspective from treating vocal pitch imitation as represented discretely across groups, to an assumption that this ability may vary in degree across individuals. Methodologically, our proposal involves a shift away from ANOVAs that incorporate quasi-experimental group variables, to the effect magnitude regression approach. We suggest that this change in perspective is more faithful to the way vocal pitch imitation is represented in the population, and that the change in methodology avoids potentially contaminating pitfalls present in the standard approach.

At the same time, we do not wish to declare spurious all past research efforts that have incorporated the standard approach, nor do we wish to prejudge those whose research needs may be better suited by the standard approach. Past research using the standard approach (including our own) likely has validity; however, it is worth re-analyzing results as has been done here with the two examples we presented. With respect to research focus, we see the proposal here as being most valuable to those who are interested in the basic mechanisms of vocal pitch imitation, as opposed to those who use measures of vocal pitch imitation to address ancillary issues. For instance, it makes sense from a research perspective to incorporate a categorical approach to vocal imitation performance if the goal is to better understand how listeners perceptually categorize singers. Likewise, a music educator who wishes to see what kind of performance accuracies predict further participation in music-related activities will likely benefit from treating performance categorically. However, for those of us who wish to understand VPID in its own right, the approach we suggest may be more fruitful.

## Conflict of Interest Statement

The authors declare that the research was conducted in the absence of any commercial or financial relationships that could be construed as a potential conflict of interest.

## Footnotes

1 It is important to note that the issue we raise here does not have to do with intrinsic properties of batteries, but instead with the use of batteries to form discrete groups. Indeed, we consider the use of standardized performance batteries to be of great use to research on VPID.

2 The interaction was statistically significant for all criteria except 30 cents.
